# A new species of the genus *Neotropiella* Handschin, 1942 (Collembola: Neanuridae) from Peru

**DOI:** 10.3897/BDJ.8.e57743

**Published:** 2020-11-25

**Authors:** José G. Palacios-Vargas, Yony T. Callohuari

**Affiliations:** 1 Universidad Nacional Autónoma de México, México, D. F., Mexico Universidad Nacional Autónoma de México México, D. F. Mexico; 2 University of Illinois at Urbana-Champaign, Urbana, United States of America University of Illinois at Urbana-Champaign Urbana United States of America; 3 Universidad Nacional Agraria La Molina, Lima, Peru Universidad Nacional Agraria La Molina Lima Peru

**Keywords:** taxonomy, chaetotaxy, Pseudachorutinae, Neotropics, Neanuridae.

## Abstract

**Background:**

*Neotropiella* is a genus of springtails which can be of medium size (2 mm) or relatively long (5 mm). These springtails live in leaf litter, under the bark of dead trees or in decomposing wood, mainly in the Neotropical Region and are often collected by litter samples on Berlese funnels or by pitfall traps. Most species have been described, based on relatively few specimens and chaetotaxy of several species is incomplete.

**New information:**

A new species within *Neotropiella* was discovered in recent pitfall trap collections from Peru. *Neotropiella peruana* sp. n. was taxonomically treated and studied under both phase contrast and scanning electron microscopy. It is similar to *N. insularis* from Brazil, but smaller with only 4 mandibular teeth (vs. 5) and with well-developed unguis lateral teeth. Intraspecific variation of the new species is provided. We also present the first DNA barcodes for the genus.

## Introduction

Members of the genus Neotropiella are often abundant in forest litter, but are also found in decomposing wood, bark and moss on branches of trees and even in epiphytic plants. They are often collected by Malaise traps, canopy fogging and by pitfall traps. The genus in mainly distributed in the Neotropical Region where 18 species have been listed (Abrantes et al. 2012, Queiroz et al. 2013). Most descriptions are very brief including the few specimens (one or two) as noted by Bellini et al. (2020) and, even in the four species for which the chaetotaxy was reported, no morphological variation was included.

The only species of this genus cited from Peru is *Neotropiella carli* (Denis, 1924) which can measure up to 5 mm long; the new species here described is less than half the size and has a stronger pigmentation and several different characters. It was collected by pitfall traps at San Ramón, Chanchamayo, fundo Génova. The *locus typicus* is Yunga lower montane forest (Fig. 1).

## Materials and methods

Material from Peru was collected by the second author using pitfall traps and preserved in 96% ethanol. A total of 19 specimens were subsequently slide-mounted in Hoyers’ solution at Laboratorio de Ecología y Sistemática de Microartrópodos, Faculty of Sciences, Universidad Nacional Autónoma de México for study.

For the Scanning Electron Microscopy (SEM) study, several specimens were dehydrated using a graduated series of ethanol dilutions, then dried using a critical point dryer (Baltec CPD030) and coated in gold using a Denton Vacuum Desk II ioniser.

For the molecular study, five specimens were photographed and sent for sequencing with the standard COI–5P marker (“DNA barcode”, Ratnasingham and Hebert 2013) at the Canadian Centre for DNA Barcoding, University of Guelph.

For the morphological study, specimens or cuticles were mounted in Hoyer’s solution after clearing the specimens. Some slides were remounted to study chaetotaxy and morphological variation.

Nineteen specimens (including vouchers and type material) were deposited in the following collections:

**LESM** Laboratorio de Ecología y Sistemática de Microartrópodos, Universidad Nacional Autónoma de Mexico, Ciudad de México, México.

**MEKRB** Museo de Entomología Klaus Raven Büller, Universidad Nacional Agraria La Molina, La Molina, Lima, Perú.

### Abbreviations used in text

A-G—labial setae of median area; Abd—abdominal segment; Ant—antennal segment; cf—cuticular fold of sensorial Ant III organ; Cx—coxa; d-f—lateral labial setae; d1-5—dorsal cephalic setae; De—dorso external area; Di—dorso-internal area; DL—Dorso-lateral area; Fe—femur; g—genal seta 4,5; hr—anal valve setae; i— dorsal microseta “i” of Ant IV; L—vestigial tuberculate labial seta L; Lb—labium; Lr— labrum; M—Median seta of tibiotarsus proximal verticile Ma—macroseta; m—microseta; m’—ventral or lateral microsensillum; m—microsetae; ms— microsensillum; Oc1, Oc2, Oc3—ocular setae 1, 2, 3; Mx—maxilla; PAO—postantennal organ; or— antenal organite; S—antennal sensillum 1-9 sensilla; Scx I—subcoxa I; Scx II—subcoxa II; sd—subdorsal seta 1-4; sf—ventral sensorial field of Ant IV; Sgd—dorsal guard sensillum; Sgv—ventral guard sensillum; ss—body sensorial setae; Tita—tibiotarsus; Th—thoracic segment; Tr—trochanter; VT—ventral tube. Setae: a—anterior row of setae; m—median row of setae; p—posterior row of setae.

## Taxon treatments

### Neotropiella peruana

Palacios-Vargas & Callohuari, 2020 sp. n.

BBD6765E-B73F-512B-9CE0-3175560DE602

BOLD:ADS2464

urn:lsid:zoobank.org:act:0D2B8C00-4652-487A-A973-58E8387FD445

#### Materials

- **Type status:** Holotype. **Occurrence:** catalogNumber: 22520; recordedBy: Y.T. Callohuari; individualCount: 1; sex: female; lifeStage: adult; **Taxon:** scientificName: Neotropiella peruana; kingdom: Animalia; phylum: Arthropoda; class: Collembola; order: Poduromorpha; family: Neanuridae; genus: Neotropiella; specificEpithet: peruana; scientificNameAuthorship: Palacios-Vargas & Callohuari; **Location:** continent: South America; country: Peru; stateProvince: Junin; county: Chanchamayo; municipality: San Ramon; verbatimLocality: Fundo La Genova; verbatimElevation: 1006 m; verbatimLatitude: -11.095869; verbatimLongitude: -75.353245; verbatimCoordinateSystem: Decimal; decimalLatitude: -11.095869; decimalLongitude: -75.353245; **Identification:** identifiedBy: Jose G. Palacios-Vargas; dateIdentified: 2018; **Event:** samplingProtocol: pitfall trap; eventDate: 28/10/2018; year: 2018; month: october; day: 28; verbatimEventDate: 28/10/2018; habitat: terrestrial; **Record Level:** language: en; institutionID: 1932; collectionID: 4001; institutionCode: FC-UNAM; collectionCode: LESM-AC; basisOfRecord: Slide mounted in Hoyers’ solution
- **Type status:** Paratype. **Occurrence:** catalogNumber: 22521; recordedBy: Y.T. Callohuari; individualCount: 1; sex: female; lifeStage: adult; **Taxon:** scientificName: Neotropiella peruana; kingdom: Animalia; phylum: Arthropoda; class: Collembola; order: Poduromorpha; family: Neanuridae; genus: Neotropiella; specificEpithet: peruana; scientificNameAuthorship: Palacios-Vargas & Callohuari; **Location:** continent: South America; country: Peru; stateProvince: Junin; county: Chanchamayo; municipality: San Ramon; verbatimLocality: Fundo La Genova; verbatimElevation: 1006 m; verbatimLatitude: -11.095869; verbatimLongitude: -75.353245; verbatimCoordinateSystem: Decimal; decimalLatitude: -11.095869; decimalLongitude: -75.353245; **Identification:** identifiedBy: Jose G. Palacios-Vargas; dateIdentified: 2018; **Event:** samplingProtocol: pitfall trap; eventDate: 28/10/2018; year: 2018; month: october; day: 28; verbatimEventDate: 28/10/2018; habitat: terrestrial; **Record Level:** language: en; institutionID: 1932; collectionID: 4001; institutionCode: FC-UNAM; collectionCode: LESM-AC; basisOfRecord: Slide mounted in Hoyers’ solution
- **Type status:** Paratype. **Occurrence:** catalogNumber: 22522; recordedBy: Y.T. Callohuari; individualCount: 1; sex: -; lifeStage: juvenile; **Taxon:** scientificName: Neotropiella peruana; kingdom: Animalia; phylum: Arthropoda; class: Collembola; order: Poduromorpha; family: Neanuridae; genus: Neotropiella; specificEpithet: peruana; scientificNameAuthorship: Palacios-Vargas & Callohuari; **Location:** continent: South America; country: Peru; stateProvince: Junin; county: Chanchamayo; municipality: San Ramon; verbatimLocality: Fundo La Genova; verbatimElevation: 1006 m; verbatimLatitude: -11.095869; verbatimLongitude: -75.353245; verbatimCoordinateSystem: Decimal; decimalLatitude: -11.095869; decimalLongitude: -75.353245; **Identification:** identifiedBy: Jose G. Palacios-Vargas; dateIdentified: 2018; **Event:** samplingProtocol: pitfall trap; eventDate: 28/10/2018; year: 2018; month: october; day: 28; verbatimEventDate: 28/10/2018; habitat: terrestrial; **Record Level:** language: en; institutionID: 1932; collectionID: 4001; institutionCode: FC-UNAM; collectionCode: LESM-AC; basisOfRecord: Slide mounted in Hoyers’ solution
- **Type status:** Paratype. **Occurrence:** catalogNumber: 22523; recordedBy: Y.T. Callohuari; individualCount: 1; sex: male; lifeStage: juvenile; **Taxon:** scientificName: Neotropiella peruana; kingdom: Animalia; phylum: Arthropoda; class: Collembola; order: Poduromorpha; family: Neanuridae; genus: Neotropiella; specificEpithet: peruana; scientificNameAuthorship: Palacios-Vargas & Callohuari; **Location:** continent: South America; country: Peru; stateProvince: Junin; county: Chanchamayo; municipality: San Ramon; verbatimLocality: Fundo La Genova; verbatimElevation: 1006 m; verbatimLatitude: -11.095869; verbatimLongitude: -75.353245; verbatimCoordinateSystem: Decimal; decimalLatitude: -11.095869; decimalLongitude: -75.353245; **Identification:** identifiedBy: Jose G. Palacios-Vargas; dateIdentified: 2018; **Event:** samplingProtocol: pitfall trap; eventDate: 28/10/2018; year: 2018; month: october; day: 28; verbatimEventDate: 28/10/2018; habitat: terrestrial; **Record Level:** language: en; institutionID: 1932; collectionID: 4001; institutionCode: FC-UNAM; collectionCode: LESM-AC; basisOfRecord: Slide mounted in Hoyers’ solution
- **Type status:** Paratype. **Occurrence:** catalogNumber: 22524; recordedBy: Y.T. Callohuari; individualCount: 1; sex: male; lifeStage: adult; **Taxon:** scientificName: Neotropiella peruana; kingdom: Animalia; phylum: Arthropoda; class: Collembola; order: Poduromorpha; family: Neanuridae; genus: Neotropiella; specificEpithet: peruana; scientificNameAuthorship: Palacios-Vargas & Callohuari; **Location:** continent: South America; country: Peru; stateProvince: Junin; county: Chanchamayo; municipality: San Ramon; verbatimLocality: Fundo La Genova; verbatimElevation: 1006 m; verbatimLatitude: -11.095869; verbatimLongitude: -75.353245; verbatimCoordinateSystem: Decimal; decimalLatitude: -11.095869; decimalLongitude: -75.353245; **Identification:** identifiedBy: Jose G. Palacios-Vargas; dateIdentified: 2018; **Event:** samplingProtocol: pitfall trap; eventDate: 28/10/2018; year: 2018; month: october; day: 28; verbatimEventDate: 28/10/2018; habitat: terrestrial; **Record Level:** language: en; institutionID: 1932; collectionID: 4001; institutionCode: FC-UNAM; collectionCode: LESM-AC; basisOfRecord: Slide mounted in Hoyers’ solution
- **Type status:** Paratype. **Occurrence:** catalogNumber: UA-467-2018; recordedBy: Y.T. Callohuari; individualCount: 1; sex: female; lifeStage: adult; **Taxon:** scientificName: Neotropiella peruana; kingdom: Animalia; phylum: Arthropoda; class: Collembola; order: Poduromorpha; family: Neanuridae; genus: Neotropiella; specificEpithet: peruana; scientificNameAuthorship: Palacios-Vargas & Callohuari; **Location:** continent: South America; country: Peru; stateProvince: Junin; county: Chanchamayo; municipality: San Ramon; verbatimLocality: Fundo La Genova; verbatimElevation: 1006 m; verbatimLatitude: -11.095869; verbatimLongitude: -75.353245; verbatimCoordinateSystem: Decimal; decimalLatitude: -11.095869; decimalLongitude: -75.353245; **Identification:** identifiedBy: Jose G. Palacios-Vargas; dateIdentified: 2018; **Event:** samplingProtocol: pitfall trap; eventDate: 28/10/2018; year: 2018; month: october; day: 28; verbatimEventDate: 28/10/2018; habitat: terrestrial; **Record Level:** language: en; institutionCode: MEKRB; collectionCode: Slides; basisOfRecord: Slide mounted in Hoyers’ solution
- **Type status:** Paratype. **Occurrence:** catalogNumber: UA-467-2018; recordedBy: Y.T. Callohuari; individualCount: 1; sex: male; lifeStage: adult; **Taxon:** scientificName: Neotropiella peruana; kingdom: Animalia; phylum: Arthropoda; class: Collembola; order: Poduromorpha; family: Neanuridae; genus: Neotropiella; specificEpithet: peruana; scientificNameAuthorship: Palacios-Vargas & Callohuari; **Location:** continent: South America; country: Peru; stateProvince: Junin; county: Chanchamayo; municipality: San Ramon; verbatimLocality: Fundo La Genova; verbatimElevation: 1006 m; verbatimLatitude: -11.095869; verbatimLongitude: -75.353245; verbatimCoordinateSystem: Decimal; decimalLatitude: -11.095869; decimalLongitude: -75.353245; **Identification:** identifiedBy: Jose G. Palacios-Vargas; dateIdentified: 2018; **Event:** samplingProtocol: pitfall trap; eventDate: 28/10/2018; year: 2018; month: october; day: 28; verbatimEventDate: 28/10/2018; habitat: terrestrial; **Record Level:** language: en; institutionCode: MEKRB; collectionCode: Slides; basisOfRecord: Slide mounted in Hoyers’ solution
- **Type status:** Paratype. **Occurrence:** catalogNumber: UA-467-2018; recordedBy: Y.T. Callohuari; individualCount: 1; sex: male; lifeStage: adult; **Taxon:** scientificName: Neotropiella peruana; kingdom: Animalia; phylum: Arthropoda; class: Collembola; order: Poduromorpha; family: Neanuridae; genus: Neotropiella; specificEpithet: peruana; scientificNameAuthorship: Palacios-Vargas & Callohuari; **Location:** continent: South America; country: Peru; stateProvince: Junin; county: Chanchamayo; municipality: San Ramon; verbatimLocality: Fundo La Genova; verbatimElevation: 1006 m; verbatimLatitude: -11.095869; verbatimLongitude: -75.353245; verbatimCoordinateSystem: Decimal; decimalLatitude: -11.095869; decimalLongitude: -75.353245; **Identification:** identifiedBy: Jose G. Palacios-Vargas; dateIdentified: 2018; **Event:** samplingProtocol: pitfall trap; eventDate: 28/10/2018; year: 2018; month: october; day: 28; verbatimEventDate: 28/10/2018; habitat: terrestrial; **Record Level:** language: en; institutionCode: MEKRB; collectionCode: Slides; basisOfRecord: Slide mounted in Hoyers’ solution

#### Description

Body length average (n = 14) 1.6 mm (range 1.25-2.25 mm). Colour in ethanol (Fig. 2): dark blue to dark purple, with yellow spots on tergites, paratergites and frontal head; eyepatches black; oral cone, legs and furcula lighter, but never white. (Fig. 2); distal antennae variably pigmented; body oval compressed dorso-ventrally; intersegments from Th I to Abd I and paratergal areas slightly developed. Microsetae 8-10 µm; macrosetae 45-50 µm; ss 50-55 µm.

**Figure 3.**
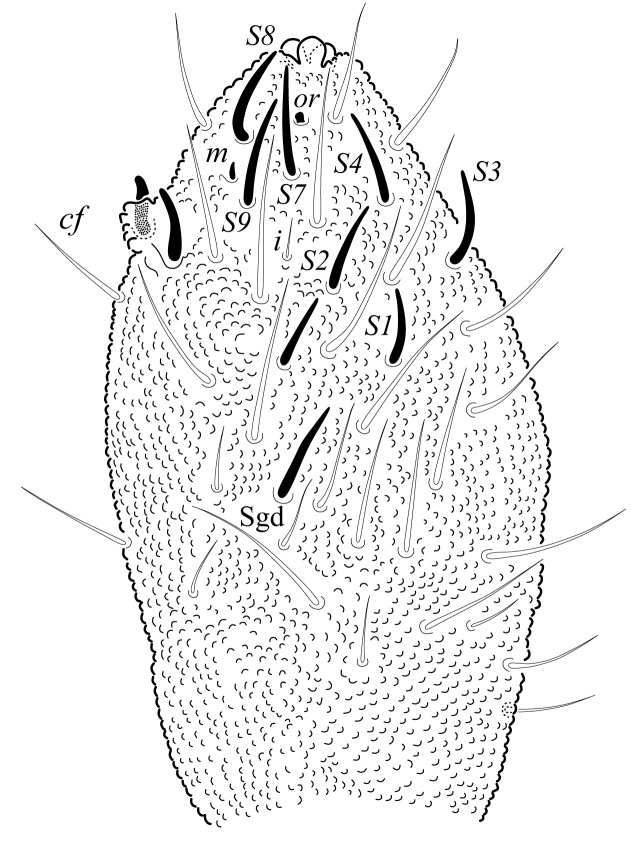
Ant III-IV dorsal view.

**Figure 4.**
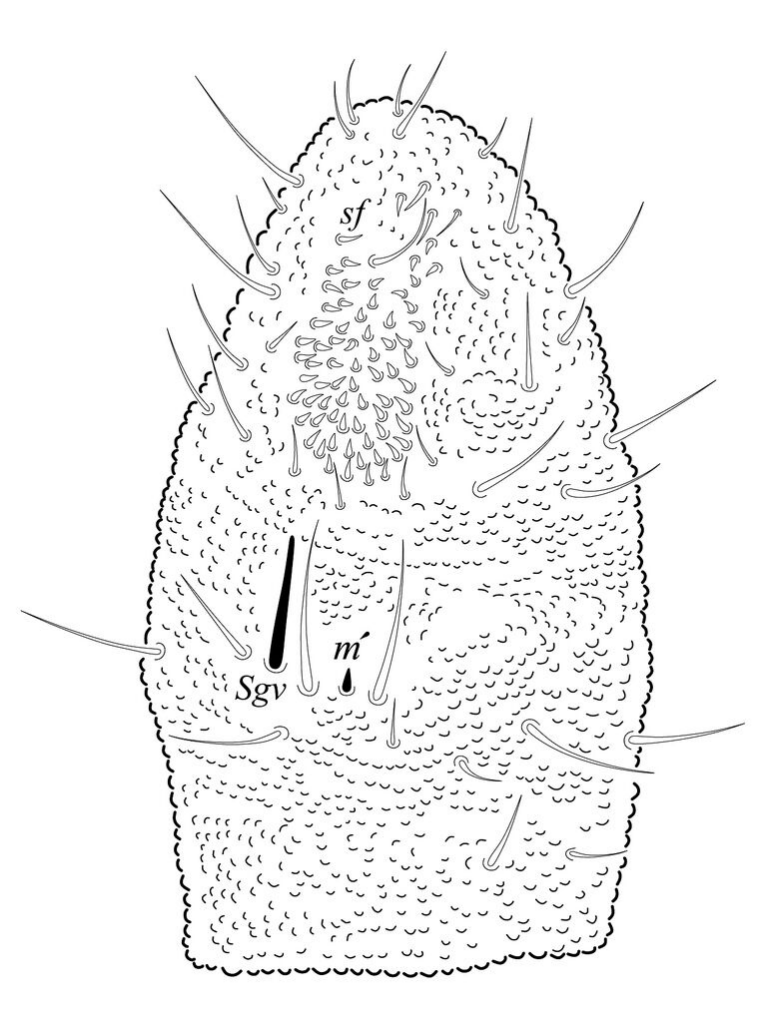
Ant III-IV ventral view.

**Figure 5.**
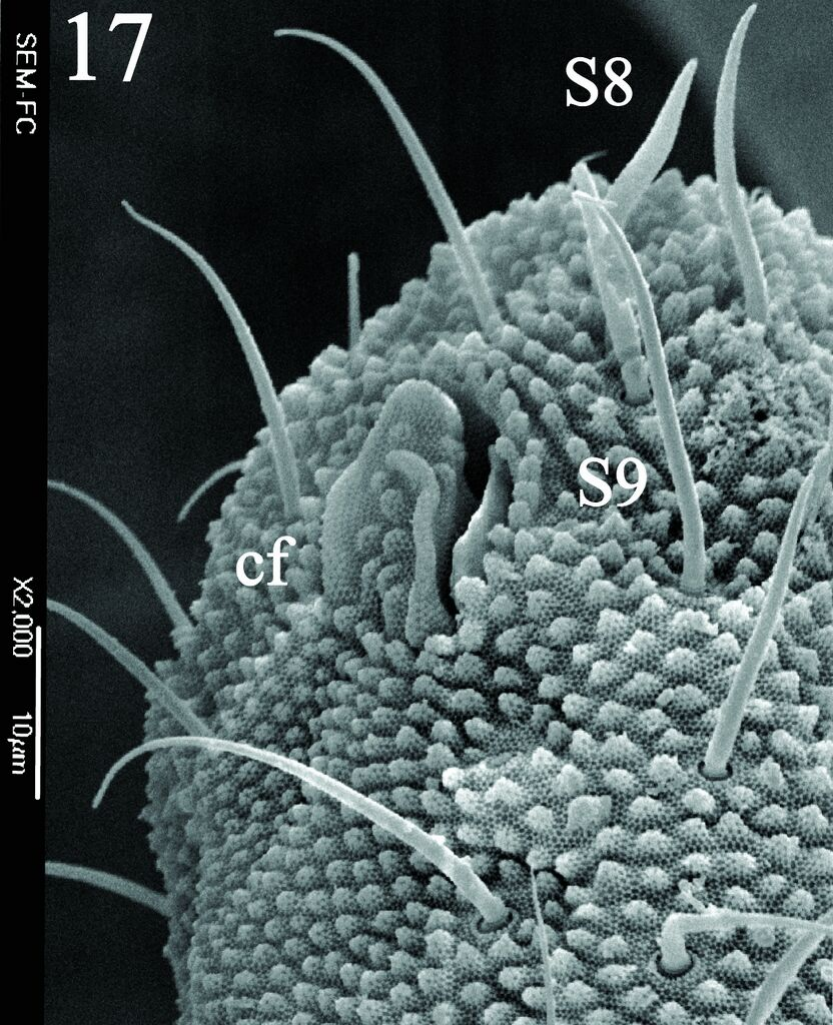
Ant III sensorial organ.

**Figure 6.**
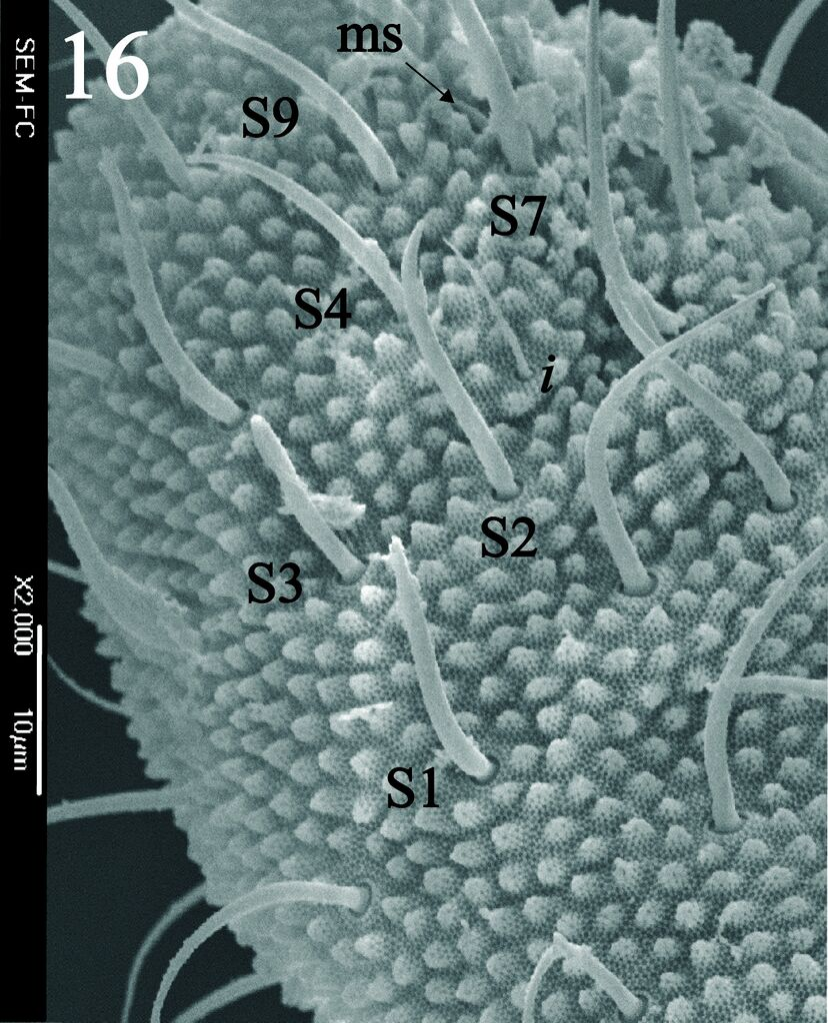
Ant IV dorsal view.

**Figure 7.**
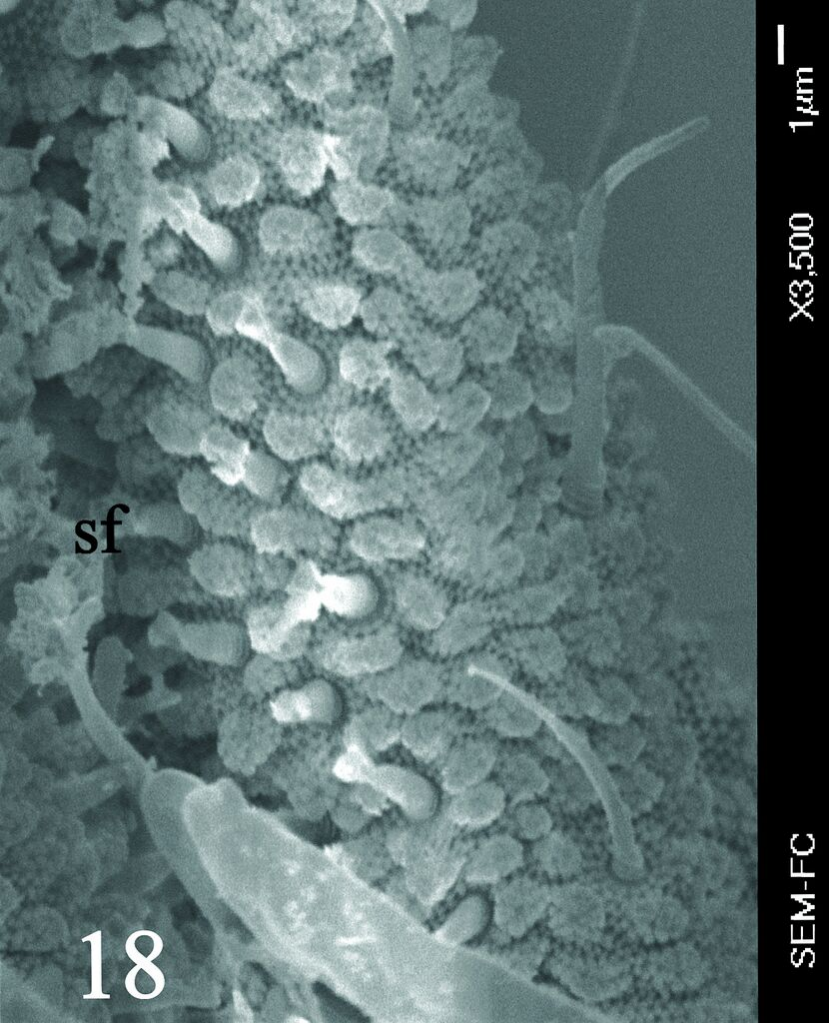
Ant. IV ventral file.

Ratio head diagonal (275 µm): antenna (300 µm) = 1:1.3. Ant I (64 µm) with 9 setae, Ant II (80 µm) with 12 setae, Ant III and IV (Fig. 3) fused dorsally (156 µm) and ventrally well separated (Fig. 4). Ratio Ant. I: II; III-IV = 1:1.1; 2.7. Sensorial organ of Ant III apically displaced to Ant IV, with 2 large tubular sensilla in a single groove slightly bending towards each other, under a large cuticular fold (Figs 3, 5), 2 longer and subcylindrical guard sensilla; Sgd thicker and longer than Sgv, ventral m’ present. Ant IV with trilobed apical bulb, 6-7 sensilla and setae “i” small and thin (Figs 3, 6), dorsolateral ms and “or” present. Ventral side of Ant IV with about 65-116 small modified setae (average 87), forming a large sensorial file that covers almost the whole ventre of segment (Figs 4, 7).

**Figure 8.**
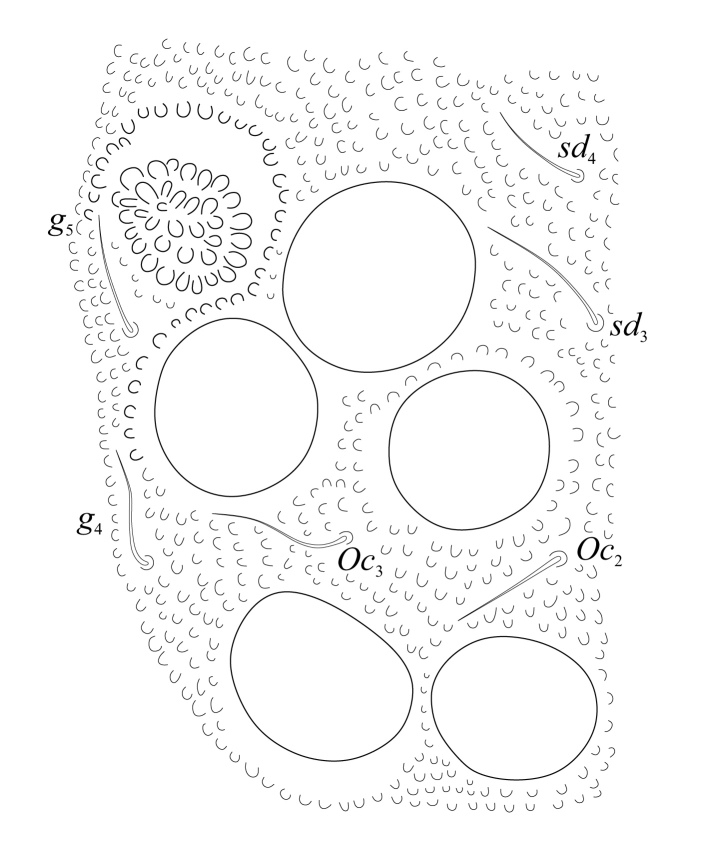
PAO and eyes.

**Figure 9.**
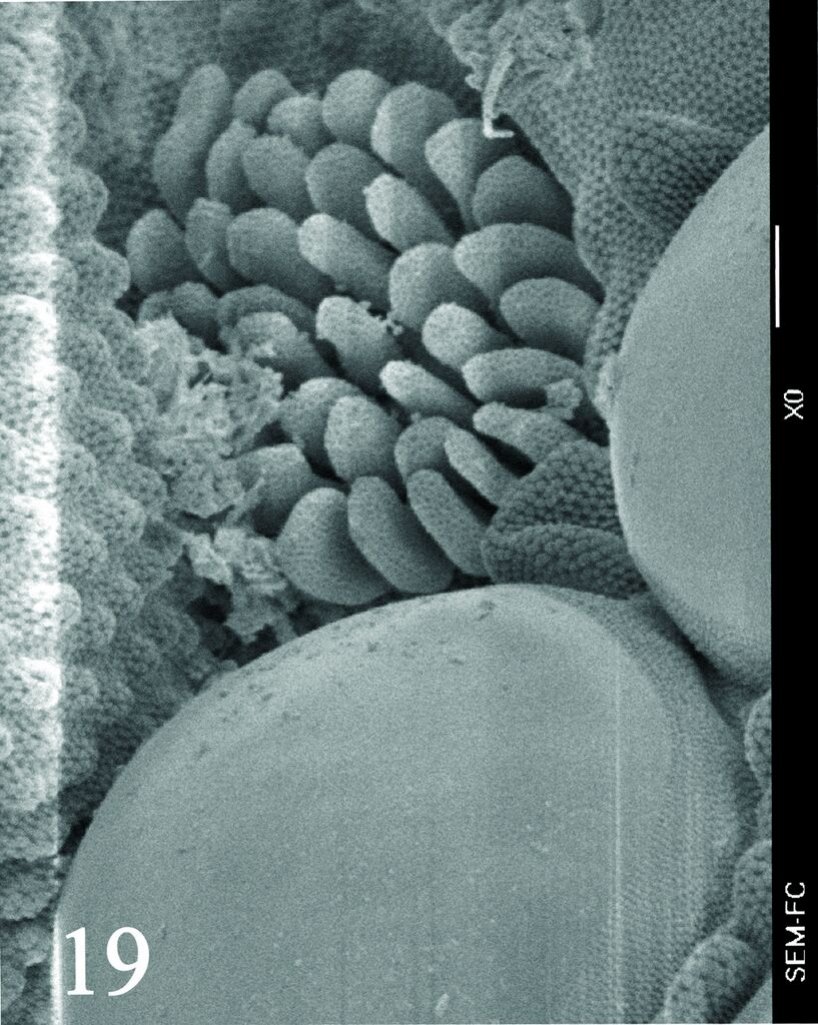
PAO and closest eyes.

**Figure 10.**
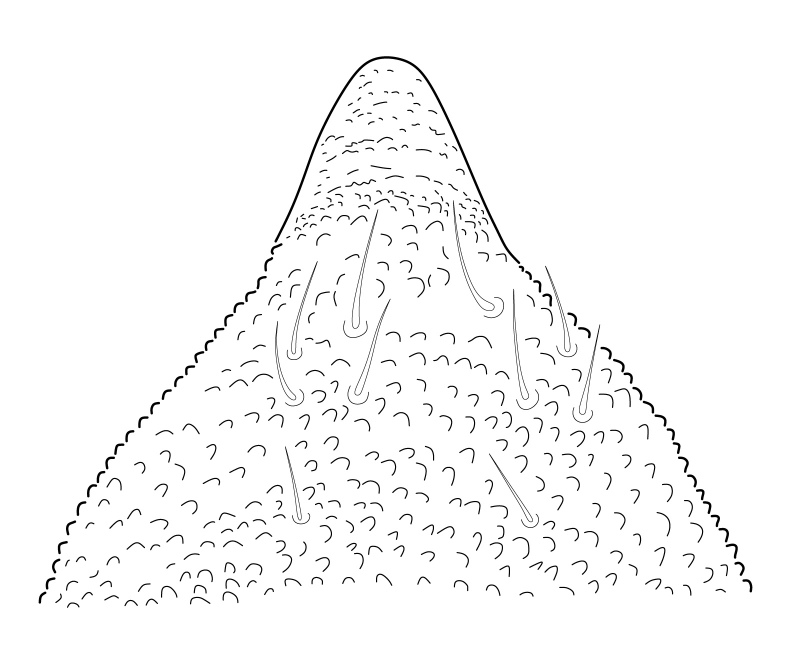
Labrum.

**Figure 11.**
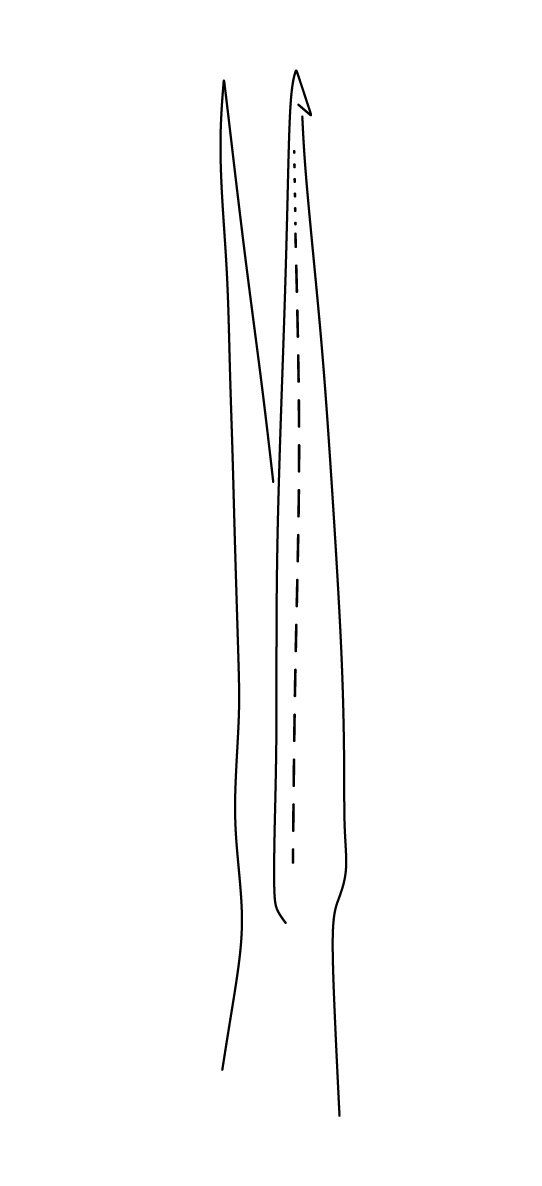
Maxilla.

**Figure 12.**
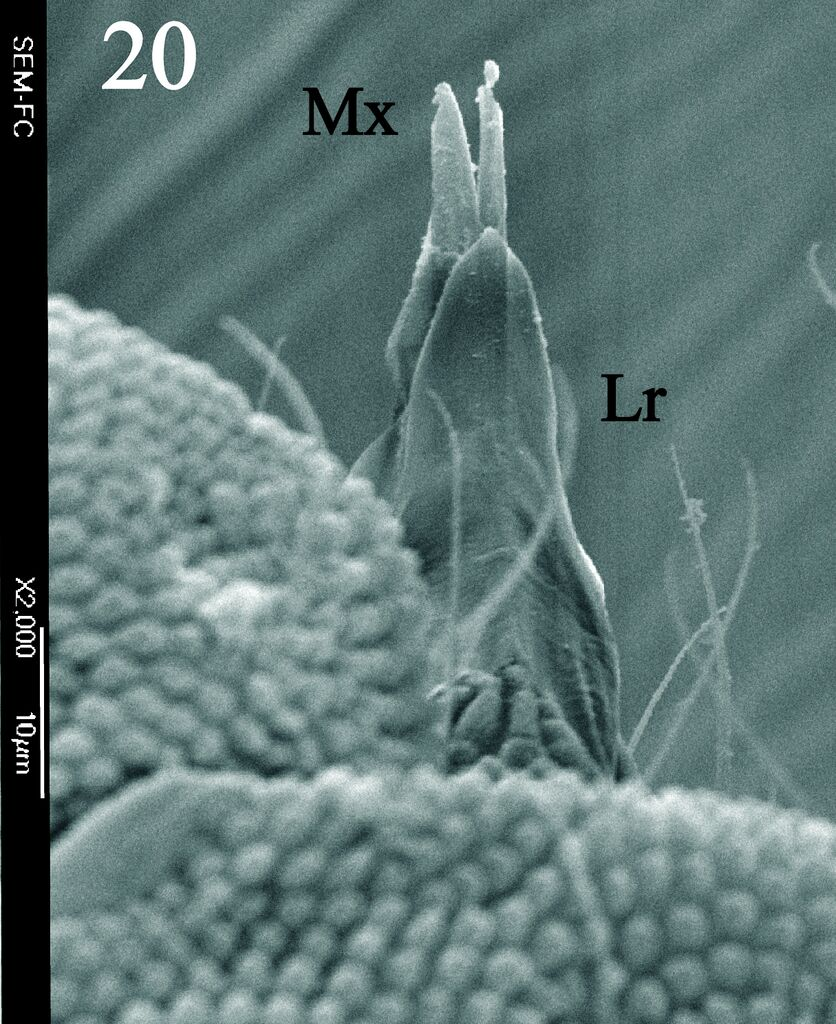
Labrum and maxilla apex.

**Figure 13.**
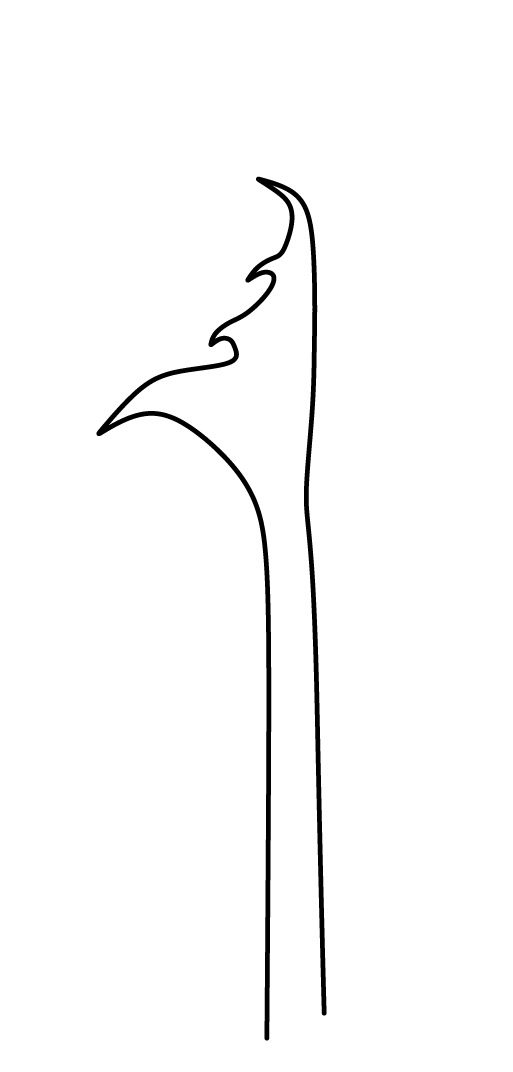
Mandible.

**Figure 14.**
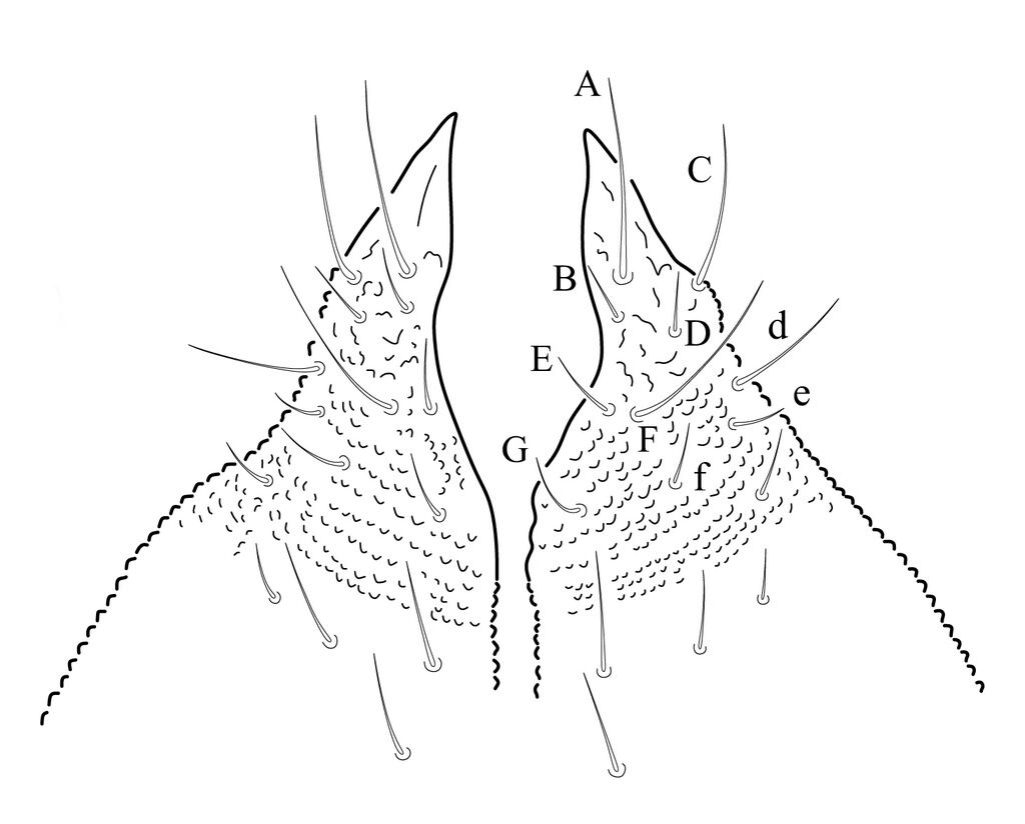
Labium chaetotaxy

**Figure 15.**
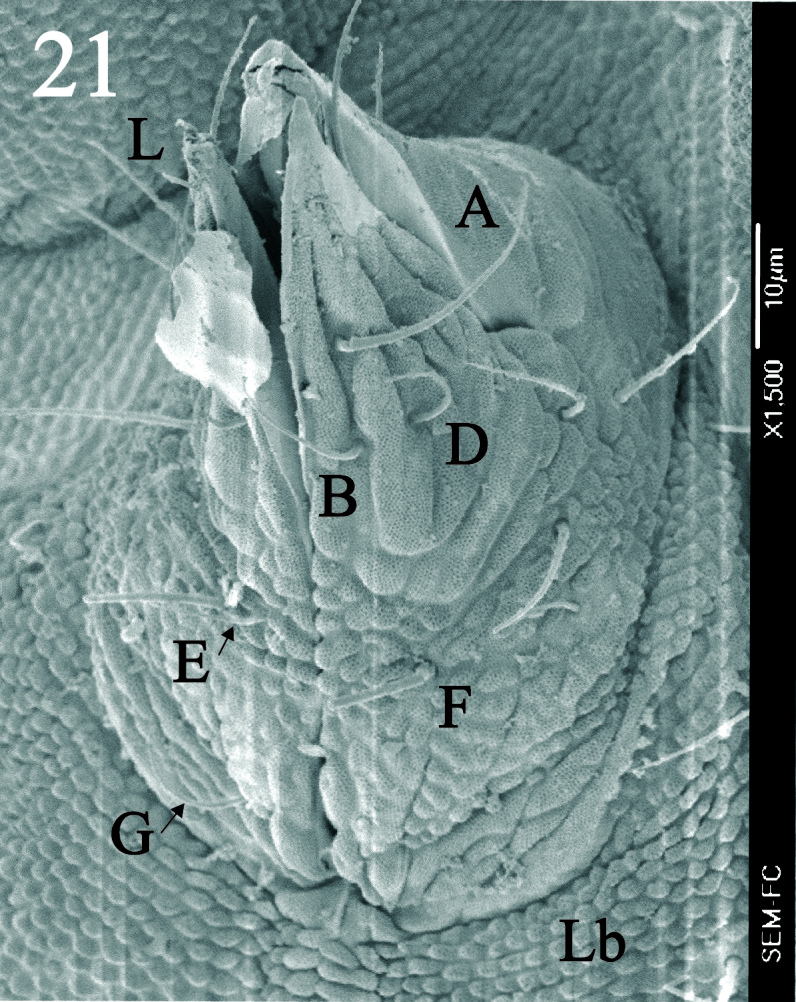
Labrum and maxilla in latero-ventral view.

Eyes 5+5 on a black eye patch (Fig. 8) about 27 - 35 µm diameter each. PAO elliptical, moruliform, slightly shorter than ocellus A (25 - 30 µm), bearing 26 – 28 simple vesicles on average (Figs 8, 9). Labrum with 0/2,4,4 setae (Fig. 10). Maxilla styliform with three lamellae, two partially fused with a small apical hook (Figs 11, 12), other acuminate with apex twisted; mandible with 4 teeth, basal tooth stronger than other teeth (Fig. 13). Buccal cone typical of the genus. Labium bearing setae A–G, (Figs 14, 15), lateral d, e and f and a tiny tuberculate apical seta L only visible under high magnification.

**Figure 16.**
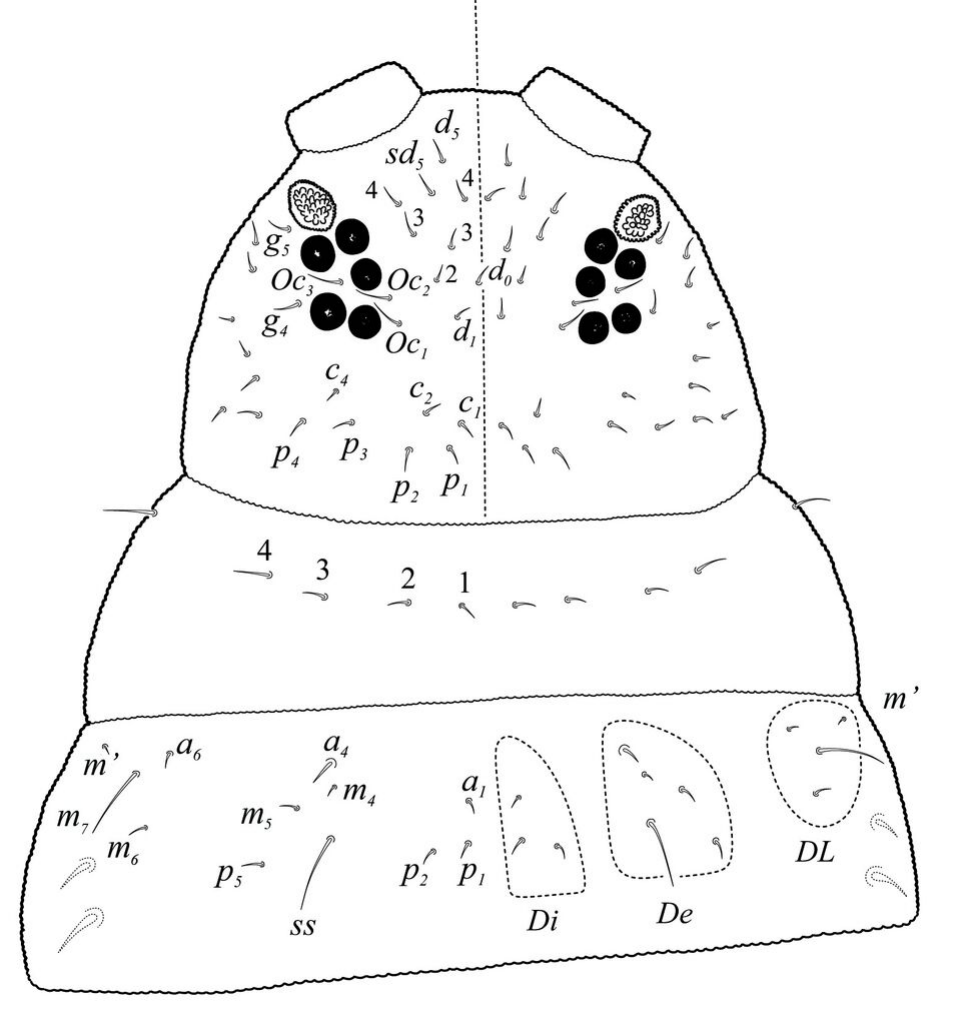
Head and thorax chaetotaxy.

**Figure 17.**
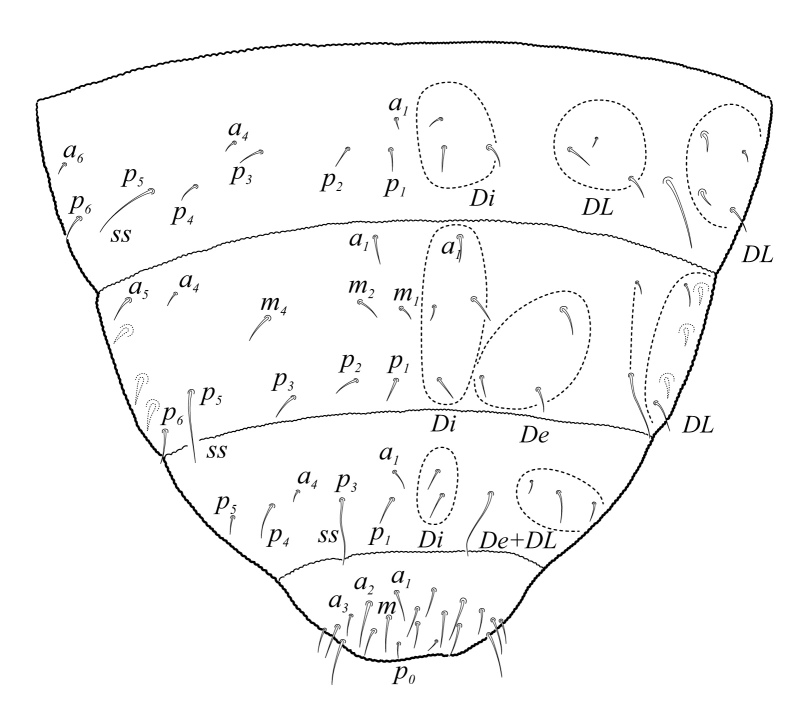
Abdominal chaetotaxy.

**Figure 18.**
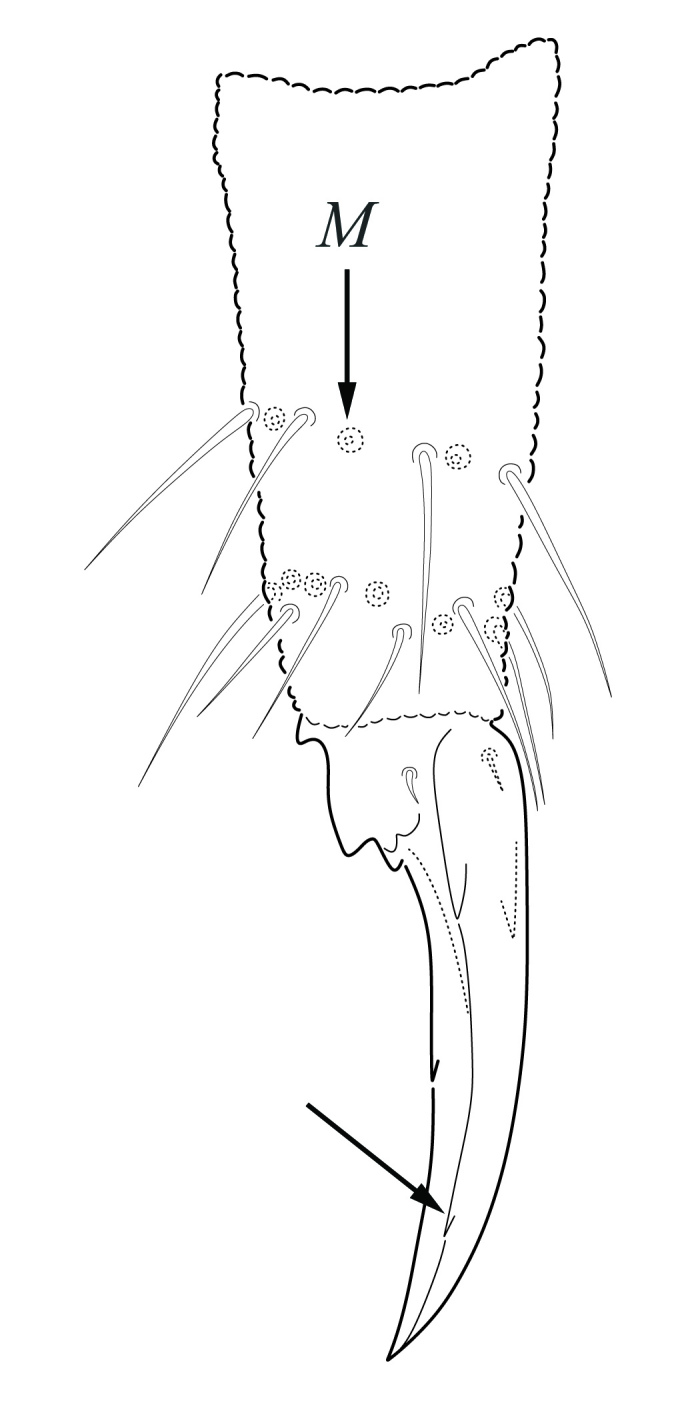
Tibiotarsus III and unguis.

**Figure 19.**
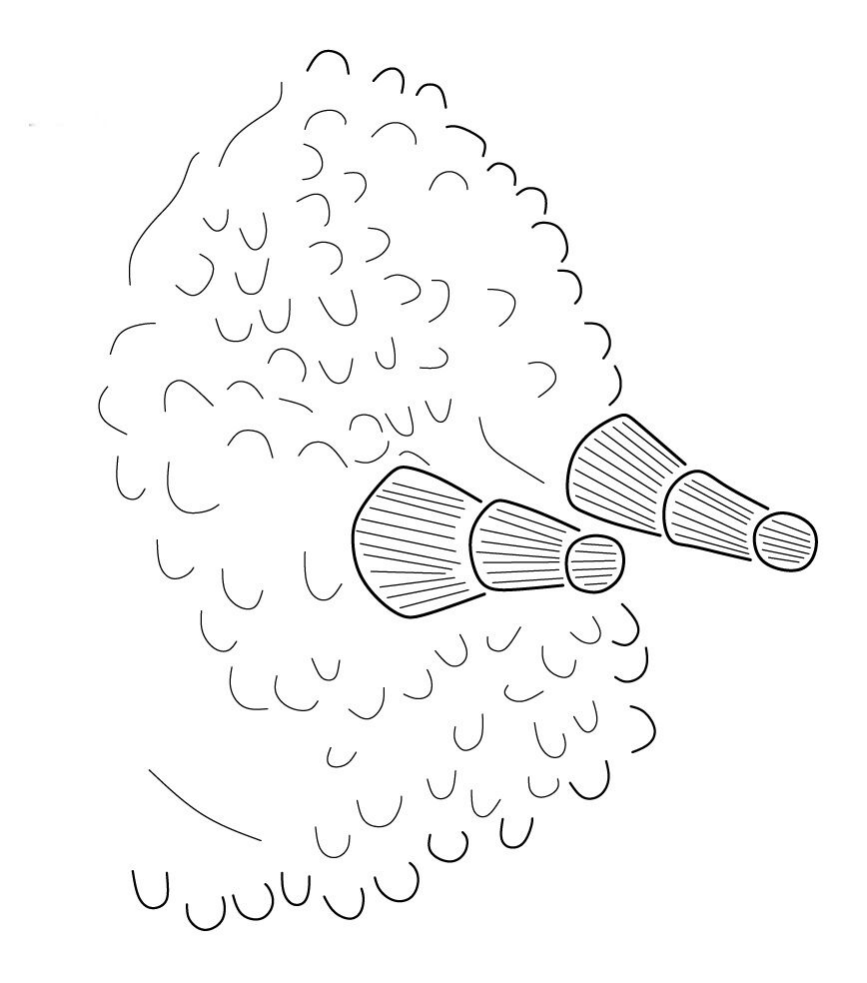
Tenaculum.

**Figure 20.**
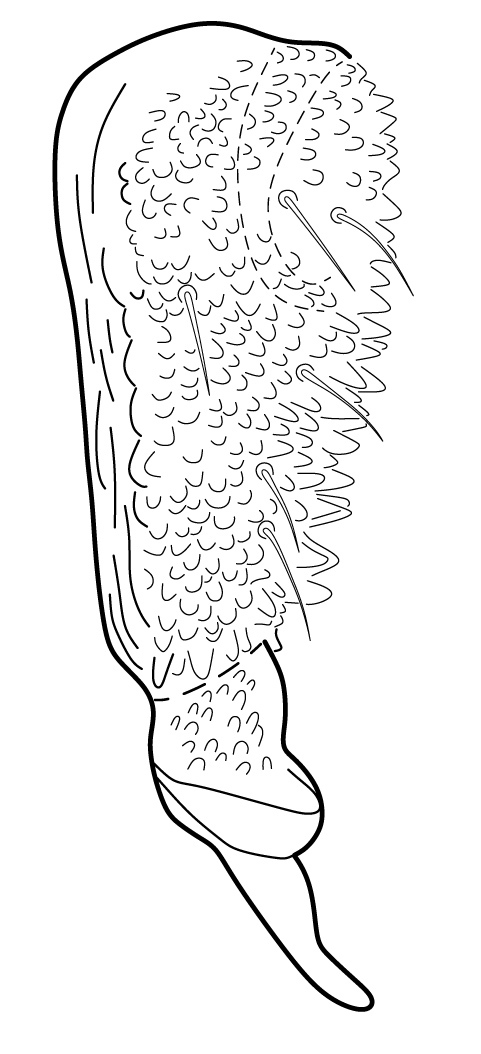
Dens and mucro, posterior view.

**Figure 21.**
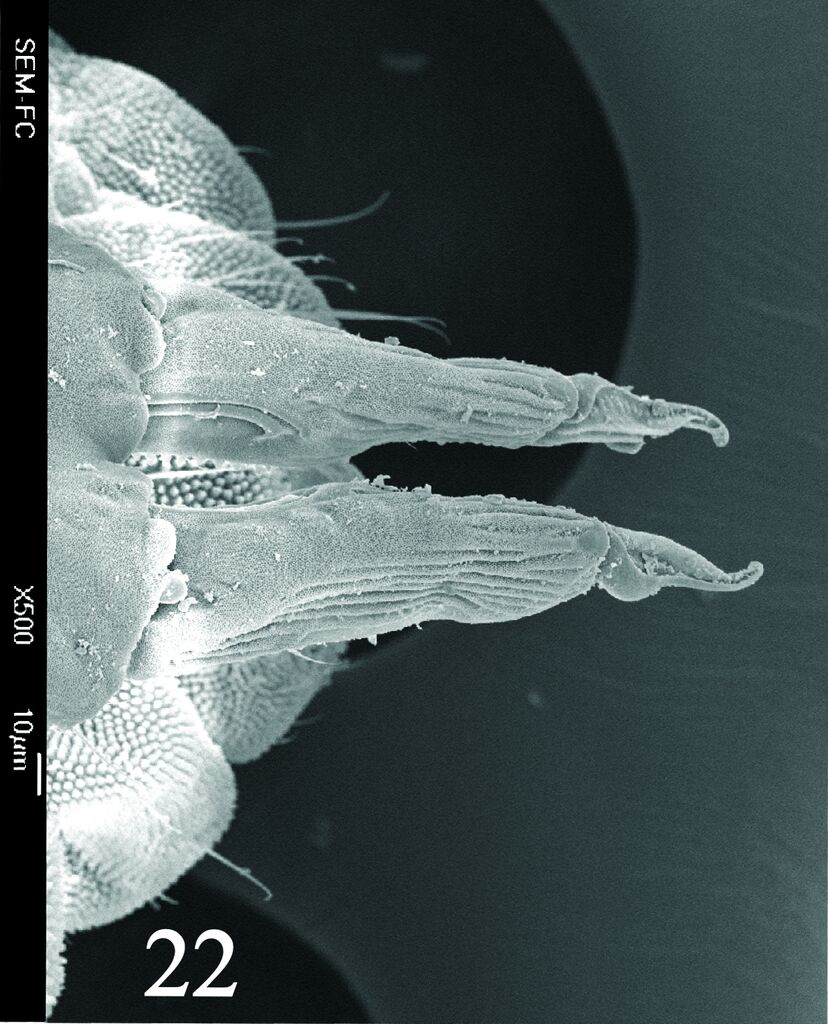
Dens and mucro, anterior view.

**Figure 22.**
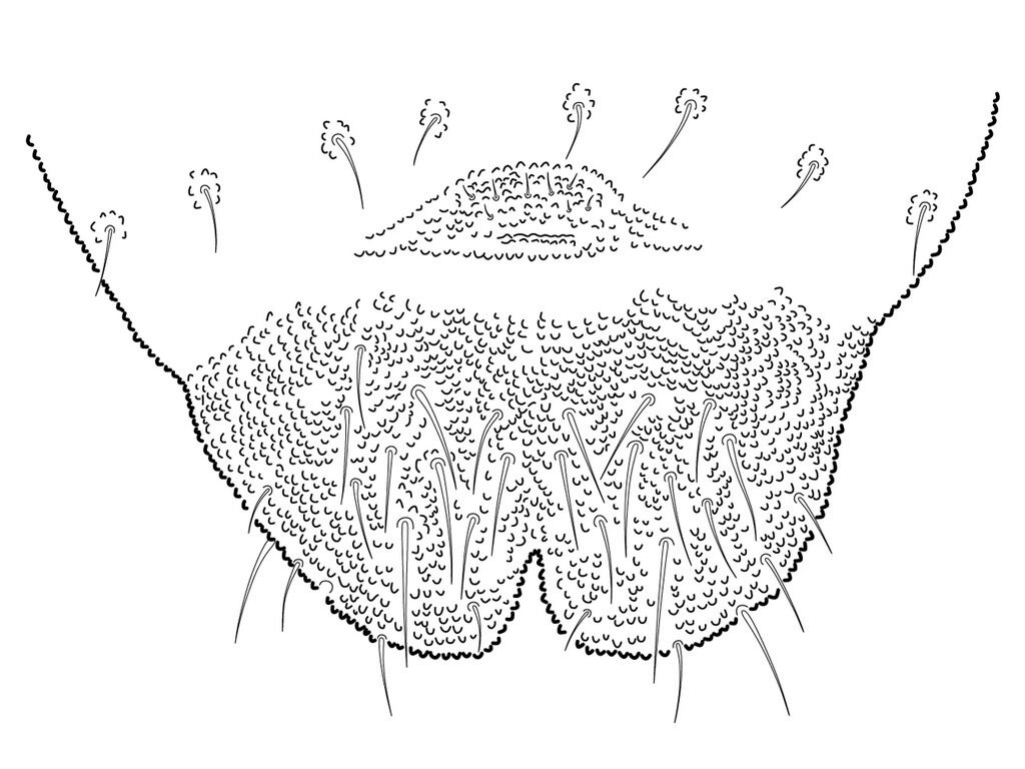
Ventral Abd. V-VI and female genital plate.

**Figure 23.**
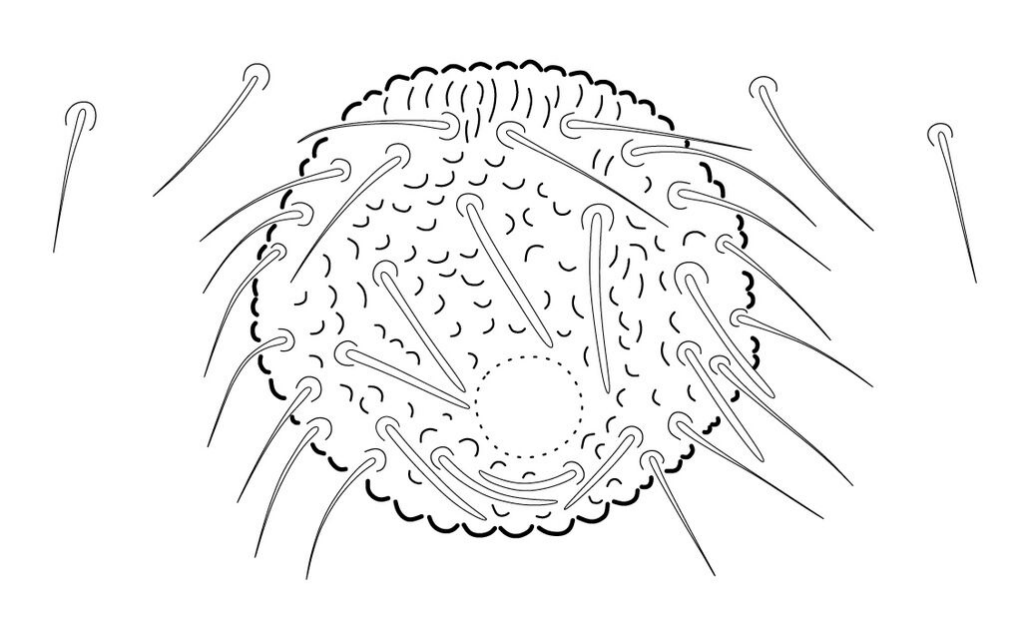
Genital plate of male.

**Figure 24.**
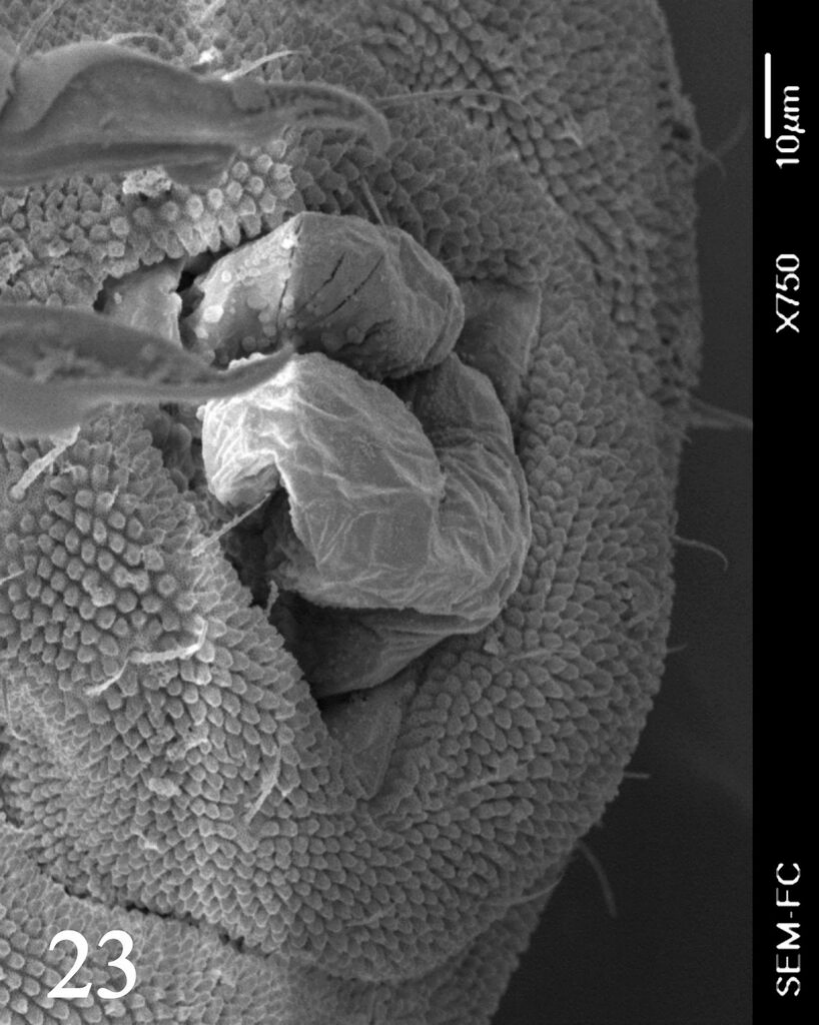
Anal vesicles.

Dorsal chaetotaxy with heterochaetosis (Figs 16, 17), composed of smooth microsetae (8-12 µm) macrosetae (45-50 µm) and long sensorial setae (50-60 µm), no plurichaetosis. Demitergite sensillar formula by half tergum from Th I to Abd VI as 022/111110. Ratio microseta: sensilla = 1: 6. Th I with 4 + 4 dorsal microsetae and 1 lateral. Th II to Abd V demitergites Di/De/DL fields with 33/33342; De 44/3333; DL 22/4445 setae, respectively. Th II–III chaetotaxy similar, with 3+3 setae on a row (a1, a4 and a6), 4+4 on m row (m4, m5–m7) and 4+4 on p row (p1, p2, p4, p5); Th. II–III sensorial sensilla as p4 and m7; lateral microsensillum (ms) present on Th II. Abd I–III chaetotaxy similar, with 3+3 setae on a row (a1, a4 and a6), 3+3 on m row (m2, m4–m7) and 5+5 on p row (p1, p4, p5, p6), sensorial setae as p5. Abd. IV chaetotaxy with 3+3 setae on a row (a1, a4, a6), 3+3 on m row (m1, m2, m4) and 5+5 on p row (p1, p2, p4, p5, p6), sensorial setae as p5. Abd V main chaetotaxy with 2+2 setae on a row (a1, a4) and 4+4 on p row (p1, p3, p4, p6), sensorial seta p3. Abd. VI with unpaired microseta p0 and no macrosetae. Chaetotaxy of legs from Scx1 to Tita: 1,2,2; 0,2,2; 2,8,7; 5,5,5; 12,11,11; 19,19,18 (proximal verticil with M seta). No clavate tenent hairs on tibiotarsi (Fig. 18); femora with a long ventral seta. Unguis with 1 inner tooth and 2 + 2 lateral moderately long teeth and sometimes a pair of tiny small distal teeth. Unguis very elongated (70-84 µm) almost as long tibiotarsus III (91 µm). Ventral tube with 4+4 setae (third pair of setae longer than others on each side). Abd II to V ventrally with 3+3, 8-9 + 8-9; 8+8; 5+5 setae. Tenaculum with three teeth on each ramus (Fig. 19). Furcula present: manubrium (187 µm) with 14-15 pairs posterior setae; dens (85-111 µm) about two times the length of mucro, with 6+6 short setae; posterior surface with strong granulation (Fig. 20), anterior surface smooth at the base and large grooves in middle and distal part (Fig. 21); mucro (40-62 µm) with two greatly developed and enlarged lamella tapering before the apex, which is elongated and slightly hooked (Figs 20, 21). Each anal valve with 13–15 setae and 2 setae hr. Genital plate of female with 3 + 3 pregenital setae, 8-10 circumgenital and 2 eugenital (Fig. 22); male with 3 + 3 pregenital, 18 circumgenital and 5 + 5 modified eugenital setae (Fig. 23). Three anal vesicles (Fig. 24).

##### Etymology

The name is locative, after of the country where it was collected in Peru.

##### Molecular results

DNA was successfully obtained from four specimens of *N. peruana* sp. n., sequences are BCICL099-19, BCICL101-19, BCICL102-19 and BCICL103-19, which were deposited in the project BCICL of the Barcode of Life Data System (http://www.barcodinglife.org/index.php). The Barcode Index Number for this species is  BOLD:ADS2464  (hyperlink activated as  https://www.boldsystems.org/index.php/Public_BarcodeCluster?clusteruri=BOLD:ADS2464).

The cuticles of four specimens were recovered and mounted in Hoyer’s solution which represent the vouchers and are kept at the author’s institution.

#### Diagnosis

*Neotropiella peruana* sp. nov. is characterised by the combination of a ventral sensorial field on Ant IV with about 100 modified setae, small body size (1.2-2.2 mm) and small number of vesicles in the postantennal organ (26-28 in average). Mandibles have 4 teeth and maxillae with 2 lamella, 1 acuminate and 1 hooked. Th I with 4 pairs of smooth microsetae and Di area on Th II and III with 3 setae. 1 + 1 lateral tooth on unguis besides the inner 1 and often 1 + 1 subapical tooth.

## Discussion

*N. peruana* sp. nov. shares with *N. carli* (Denis 1924) from Guyana and *N. insularis* Queiroz et al. (2013) from Brazil the presence of a ventral sensorial field on Ant IV with about 100 modified setae, but differs from *N. carli* in being much smaller (1.2-.2.2 vs. 4.3-6.0 mm) and having fewer vesicles in the postantennal organ (26-28 on average, vs. 50-65). *N. insularis* is also larger than *N. peruana* sp. nov. (2.5-3.0 vs. 1.2-2.2 mm), with 5 mandibular teeth (vs. 4) and no lateral teeth on unguis (vs. 1-2 pairs). The body chaetotaxy is very similar in both species, Th I with 4 pairs of smooth microsetae and Di Tubercle on Th II and III with 3 setae, but De tubercle of *N. insularis* has only 2 microsetae on the dorso-lateral area of Th II and III (vs. 3), and there is also one extra pair of setae between Abd. III and IV which is lacking in *N. peruana* sp. nov. *N. insularis* was characterised by Queiroz et al. (2013) as having composed vesicles in PAO. Lawrence (1971) suggested the dubious value of vesicles for splitting species, based on a short series and he noted that specimens with a small number of vesicles have relatively larger size, showing a tendency to subdivide; the composed PAO that was supposed to be a differential character of *N. insularis* is variable and has no importance for the species delimitation.

**Variation**: For body length, only 14 specimens were measured (adults and juveniles) from the same locality. The average is 1.665 µm (range 1.250-2.250 µm). Ant IV sensilla have usually 6, but few specimens had 7-8 or even 5. Ant IV ventral file, from 65 to 116 modified setae (average 87). Eyes are mostly constant, 5 + 5, but in one case, there was one specimen with 5 + 4 eyes. PAO, minimum vesicles 20, maximum 53 vesicles, average 26-28. Mandibles always with 4 teeth. Maxilla always hooked. Labium always the same pattern, only one specimen had 0 + 1 postlabial seta. Unguis was studied in 17 specimens; they always had one inner tooth and 2 lateral teeth, but in 7 cases, there was one pair of subapical small teeth as shown in Figure 8. Head chaetotaxy seems to be constant. Body chaetotaxy variation was studied in 14 well preserved specimens, but only on Th. I and Abd. V was strong variation found. The seta m1 of Th I is always microseta (14 cases); m2 as microseta in 3 cases and as meso- or macroseta in 11 cases; m3 as microseta in 4 cases and meso- or macroseta in 8 cases; m4 is always microseta; lateral seta is always microseta. On Abd V, the seta p1 is always microseta; p3 is always the sensorial seta, p4 is always a microseta, seta p5 is microseta, in one case, it was macroseta, lateral seta p6 is always microseta. One specimen has one teratological antenna, where only the basal segment was present and at the tip one large sensillum, another has one teratology on leg II where the tibiotarsus was lacking. One female presents only 1 eugenital seta instead of 2 such setae.

## Supplementary Material

XML Treatment for Neotropiella peruana

## Figures and Tables

**Figure 1a.**
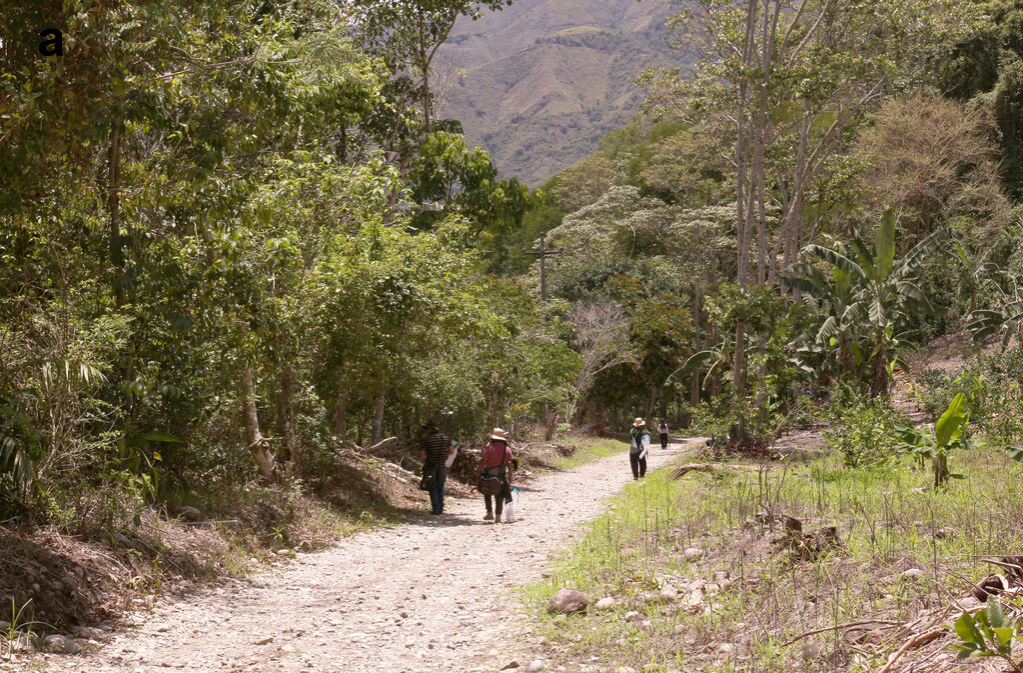


**Figure 1b.**
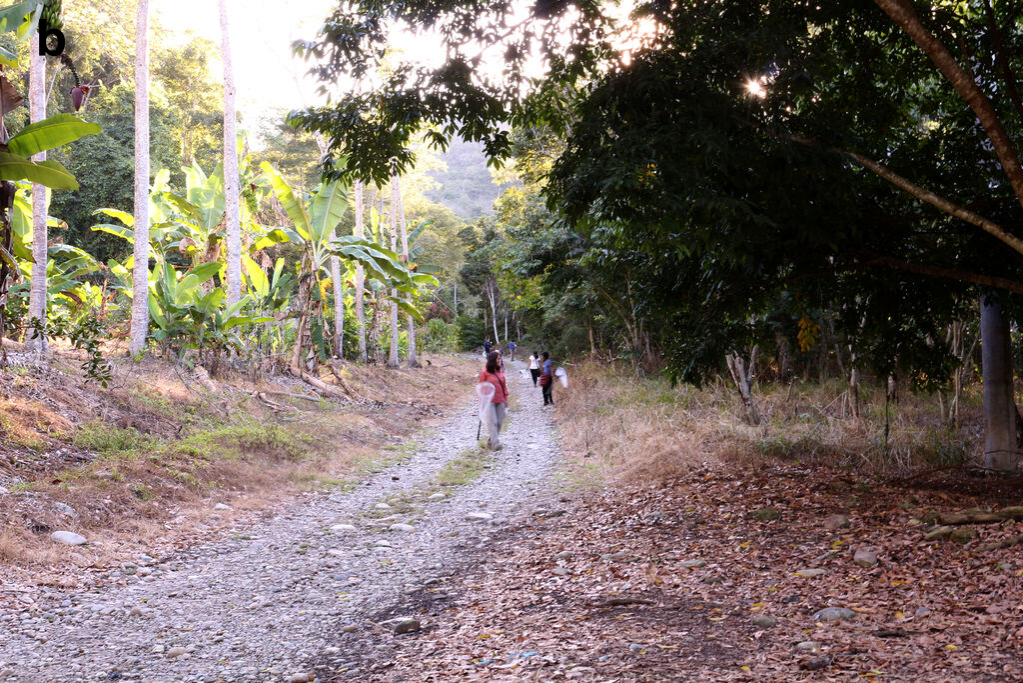


**Figure 2a.**
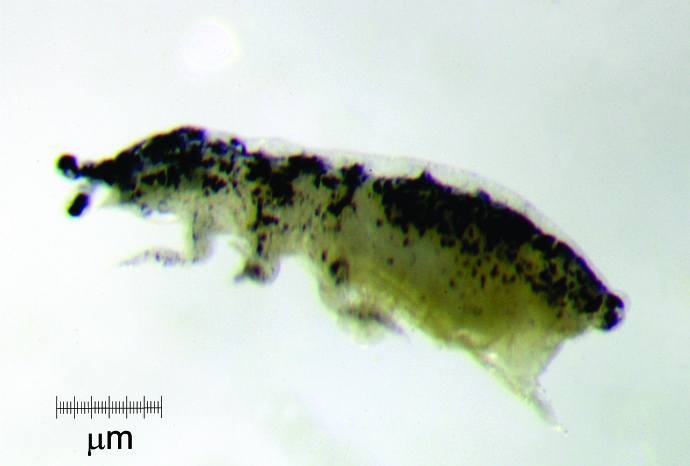
Habitus

**Figure 2b.**
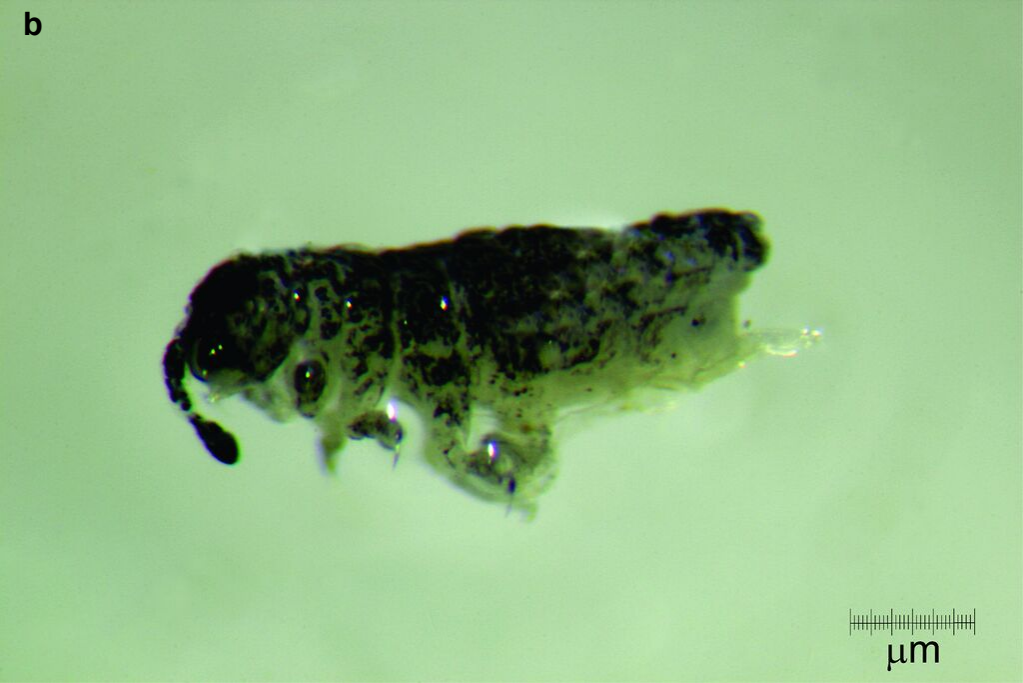
Habitus
